# Concomitant fibromyalgia complicating chronic inflammatory arthritis: a systematic review and meta-analysis

**DOI:** 10.1093/rheumatology/key112

**Published:** 2018-05-16

**Authors:** Stephen J Duffield, Natasha Miller, Sizheng Zhao, Nicola J Goodson

**Affiliations:** 1 Department of Musculoskeletal Biology 1, Institute of Ageing and Chronic Disease, University of Liverpool, Liverpool, UK; 2 Clinical Sciences Centre, University Hospital Aintree, Liverpool, UK; 3 School of Medicine, University of Liverpool, Liverpool, UK

**Keywords:** fibromyalgia, rheumatoid arthritis, ankylosing spondylitis, psoriatic arthritis, comorbidity, spondyloarthritis, systematic review, meta-analysis, disease activity scores

## Abstract

**Objectives:**

This systematic review and meta-analysis will describe the prevalence of concomitant FM in adults with inflammatory arthritis and quantify the impact of FM on DAS.

**Methods:**

Cochrane library, MEDLINE, Psychinfo, PubMed, Scopus and Web of Science were searched using key terms and predefined exclusion criteria. As appropriate, proportional and pairwise meta-analysis methods were used to pool results.

**Results:**

Forty articles were identified. In RA the prevalence of FM ranged from 4.9 to 52.4% (21% pooled). In axSpA the range was 4.11–25.2% (13% pooled in AS only). In PsA the range was 9.6–27.2% (18% pooled). The presence of concomitant FM was related to higher DAS in patients with RA and AS (DAS28 mean difference 1.24, 95% CI: 1.10, 1.37 in RA; BASDAI mean difference 2.22, 95% CI: 1.86, 2.58 in AS). Concomitant FM was also associated with higher DAS in existing PsA studies. Self-reported, rather than objective, components of DAS appear to be raised in the presence of FM (e.g. tender joint count and Visual Analogue Scale (VAS) pain scores).

**Conclusion:**

FM is common in RA, AxSpA and PsA. Comorbid FM appears to amplify DAS and could therefore influence management of these rheumatic conditions.

**Rheumatology key messages**

- Fibromyalgia is very common in chronic inflammatory arthritis, compared with prevalence in the general population.
- Comorbid fibromyalgia may influence disease activity scores, giving rheumatologists an inaccurate impression of disease severity.
- Cautiously interpret disease activity indices, considering objective clinical measurements, in patients with comorbid fibromyalgia.

## Introduction

FM syndrome is a complex neurosensory disorder characterized by a history of diffuse and persistent musculoskeletal pain, with numerous discrete tender points elicited on clinical examination [1, 2]. Additional symptoms such as fatigue, sleep disturbances and anxiety are associated features of this syndrome [2, 3]. FM is strongly associated with female gender [4] and age [5] and has a prevalence of around 1–5% in the general population [6, 7].

FM is often considered a diagnosis of exclusion, but patients with inflammatory arthropathies commonly meet the criteria for FM [8], a phenomenon known as fibromyalgianess [9]. The exact prevalence of this concomitant FM in inflammatory arthropathy is debated [10, 11]. It is also unclear whether FM arises as a complication of the index condition or occurs independently in susceptible individuals. Regardless of the underlying aetiology, the presence of concomitant FM and its impact on the underlying inflammatory condition may be important. Modern rheumatological management is increasingly target driven, involving escalation of drug therapy in order to achieve optimum reduction in disease activity or disease remission [12]. Assessing disease activity in chronic inflammatory arthritis relies, in part, on self-assessment by the patient [13, 14]. Consequently, FM, which causes patients to experience pain independent of the inflammatory processes, may lead to inflated disease activity measures and, therefore, to inappropriate escalation, or inappropriate stopping, of treatment in the underlying index rheumatic condition.

The primary aim of this systematic review is to report the prevalence of FM in adult patients with chronic inflammatory arthritis. The secondary aim is to compare DAS between those with and without FM within these index conditions, and thereby assess the impact of comorbid FM on disease activity assessment.

## Methods

A systematic review following the Preferred Reporting Items for Systematic Reviews and Meta-Analyses guidelines (PRISMA) [15] was undertaken. Cochrane library, MEDLINE, Psychinfo, PubMed, Scopus and Web of Science were searched independently by two reviewers (N.M., S.J.D.) on 30 November 2017 using search terms in the following algorithm: (rheumatoid arthr* or spondyloarthr* or ankylosing or psoriatic) and FM and (prevalen* or frequency or disease activity). The wildcard function was employed to include similar terms. The protocol was registered in the International Prospective Register of Systematic Reviews (PROSPERO) in advance of completion of this work (registration number CRD42017076504).

Studies were included if they: were published in the English language; included patients with a pre-existing diagnosis of RA, AxSpA or PsA (diagnosed using recognized and validated criteria); stated the number or percentage of patients in their study diagnosed with FM (according to either 1990 or 2010 ACR criteria) or reported the impact of comorbid FM upon DAS; and were available in full text. Authors were contacted in cases where we were unable to find the full paper. Reviews, comments and editorials were excluded.

The authors independently extracted relevant data from the included articles into predefined tabulated summaries. These data included: classification systems used to diagnose the index condition; important characteristics of the study patients (gender, age and disease duration); and, if available, the prevalence of comorbid FM, or DAS for patients with and without comorbid FM. A standardized quality assessment score, the Newcastle–Ottawa Scale, was used to assess for bias at the study level. In cross-sectional studies a modified version was used [16]. For these assessments, the included studies were assigned up to 10 points based on the quality of the methods used across three domains: selection of study participants; spread of confounders (age, gender, disease duration) between the comparison groups; and ascertainment of exposure. Where a study only contributed information on prevalence, and not disease severity, quality was assessed using only the Newcastle–Ottawa Scale criteria for sample representativeness, sample size justification, comparison with non-respondents and ascertainment of the exposure (presence of comorbid FM). These studies were assigned up to 5 points.

To provide a meaningful summary across each of the index conditions, meta-analysis was considered only when a group of studies were sufficiently homogeneous in terms of index disease classification, FM criteria and outcome criteria (e.g. type of DAS). Articles that pre-selected numbers of participants with FM, such as in case–control design, were not included in meta-analysis of prevalence. Meta-analysis of prevalence was performed using Metaprop proportional meta-analysis tool in Stata 14 (StataCorp, College Station, TX, USA). Prevalence estimates were reported as percentages. Where possible, DAS were also pooled to find an overall estimate of the additional impact of comorbid FM. These were reported as overall mean differences with 95% CIs. Meta-analysis of DAS was performed using Review Manager (RevMan) version 5.3 (The Nordic Cochrane Centre, The Cochrane Collaboration, Copenhagen, Denmark). The statistical heterogeneity of meta-analysis estimates were assessed using the *I*^2^ statistic (Der Simonian-Laird). Results were pooled using random effects methods when statistical heterogeneity was high (*I*^2^ > 75%). Forrest plots were produced in order of sample size in order to assess risk of publication bias. In the case of high statistical heterogeneity, meta-analysis was stratified by important study-level variables such as sample-size, country, study risk of bias and sample selection methods in order to explore causes of between-study variability across reported effect estimates.

## Results

**Fig. 1:**
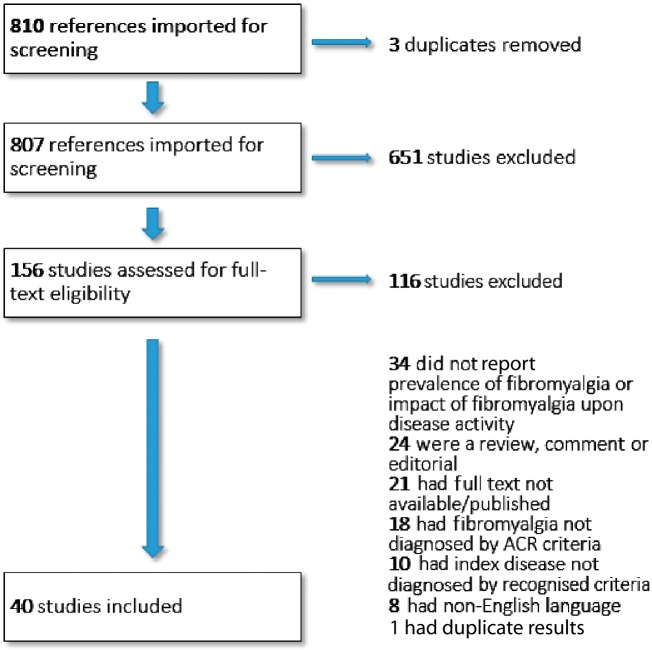
Flowchart showing selection of suitable articles

A total of 810 articles were generated through the database searches. These were screened for eligibility using the titles and abstracts. A further 651 articles were excluded as irrelevant. The full-texts of the remaining 156 articles were then sought and assessed for eligibility. Thirty-four articles were excluded for not reporting the prevalence of FM, or the impact of FM upon disease activity, in the index conditions of interest. Eighteen studies were excluded for having divergent or unclear definitions of FM (1990 or 2010 ACR criteria were required). Ten further articles were excluded for not clearly using validated diagnostic classifications for RA, AS, AxSpA or PsA. Twenty-one articles were excluded as the full-text had not been published. Twenty-four articles were excluded due to being reviews, comments or editorials. Eight articles were excluded due to being non-English language. Lastly, one study reported duplicate results from a cohort of patients recruited in another included study. Figure 1 shows the PRISMA flowchart.

In total, 40 articles met the inclusion criteria [11, 13, 14, 17–53]. Of the included studies, 29 reported data on the prevalence or impact of FM in RA [11, 14, 17–41, 52, 53]. Nine articles studied AxSpA, with eight articles focusing on FM in AS [11, 13, 27, 45, 47–50], and two papers looking at non-radiographic axial spondyloarthritis [27, 45]. Lastly, six studies focused on PsA patients [27, 42–44, 50, 51].

The majority of studies were based in single rheumatology departments or clinics [11, 14, 17, 18, 20–22, 24–29, 31–33, 35, 36, 37, 38, 40, 41, 43, 46–50, 52], some studies used participants from multiple sites or centres [30, 34, 39, 42, 53], and some had many centres contributing to a single registry or database [13, 19, 23, 45]. Study setting also varied by country with five each from the UK [14, 32, 34, 36, 45] and the USA [18, 19, 31, 39, 53], four each from Brazil [21, 22, 37, 18] and Italy [42, 43, 50, 52], three from Egypt [26, 28, 35], France [24, 27, 46] and Turkey [11, 20, 49], two from Denmark [23, 33], Israel [47, 51], Spain [13, 40] and Pakistan [17, 41], and one each from Australia [30], Canada [44], India [25], Netherlands [38] and Romania [29].

Newcastle–Ottawa quality assessment scores can be found in supplementary Tables S1 and S2, available at *Rheumatology* online. For cross-sectional studies reporting disease activity, the quality ranged from 3 to 8 out of 10 possible points (mean score 5.5); quality was higher in case–control studies, ranging from 7 to 8 out of 10 (mean 7.6). Among studies reporting only prevalence scores, quality scores ranged from 2 to 4 out of 5 possible points (mean 3.1). Most included studies were at least moderate, if not high quality. As described above, all included studies used recognized criteria to diagnose both the index condition and FM exposure in their study participants. Among the studies that reported prevalence alone, common reasons for lower scoring included unjustified sample size and failure to described eligible participants who did not take part. Amongst the studies that reported disease activity, common reasons for lower scoring were poor description of statistical methods, non-blinding of examiners and important differences between comparison groups. Gender, age and disease duration were well reported in the included studies and 19 papers were assessed as lower quality due to differences between comparison groups for at least one of these factors [11, 13, 14, 20, 22, 24, 34, 36, 37, 38, 40, 43, 46–52]. Other confounding factors may have been important, but amount of comorbidity and treatment regimen were reported only in a small number of studies. In the reporting studies, the difference in types of anti-inflammatory drugs taken between those with and without FM was generally found to be non-significant [13, 17, 20, 21, 24–26, 29, 46]. On the other hand, mental health scores for anxiety or depression, or number of participants on regular antidepressants, were significantly worse in the FM group in more than half of the reporting studies [14, 24, 34, 35, 37, 40].

Summary details, patient characteristics and prevalence of FM in included studies can be found in the tabulated summaries in the supplementary data. See supplementary Tables S3–S5, available at *Rheumatology* online, for RA, AS/axSpa and PsA, respectively. Supplementary Tables S6–S8, available at *Rheumatology* online, compare data on DAS for those with and without concomitant FM in RA, AS/AxSpA and PsA, respectively.

### RA

Twenty-nine studies described the prevalence of FM in RA patients. Extracted data was cross-sectional apart from in three case–control studies [21, 29, 35], from which it was not possible to assess prevalence. In addition, one study recruited participants until an equal number of people with and without comorbid FM had been recruited, and thus any prevalence figures from this study would be meaningless [34]. It was possible to determine an estimate of comorbid FM prevalence from the other 25 RA studies. Many articles additionally assessed the relationship between FM and disease activity [14, 17, 20–22, 24–26, 29, 32–36, 37, 38, 40, 41, 52]. The prevalence of FM in patients with RA varied considerably from 4.9% [27] to 52.4% [28]. In proportional meta-analysis, the overall prevalence rate of FM was 21% (95% CI: 17, 25%) across all studies (see supplementary Fig. S1, available at *Rheumatology* online).

Heterogeneity was high between studies for meta-analysis of prevalence (*I*^2^ = 92.2%). However, when studies were put in order of sample size (supplementary Fig. S1, available at *Rheumatology* online) the larger studies reported more consistent and lower estimates for prevalence in RA. For example, including only studies with larger sample sizes (*n* > 150) brought pooled estimate of prevalence down to 14% [10–18] though this only improved heterogeneity slightly (*I*^2^ = 90.5%). Forest plots showed no evidence of publication bias.

There was good consistency across the 19 unique studies reporting data on the impact of FM upon DAS28 and its components. All but one study reported higher DAS28 in participants with comorbid FM. Sixteen of these found statistically significant increased DAS in RA patients with comorbid FM compared with those without (see supplementary Table S2, available at *Rheumatology* online) [14, 17, 21, 22, 24–26, 29, 32–35, 37, 38, 40, 41]. Furthermore, these patients had significantly higher tender joint counts [14, 17, 21, 22, 24, 25, 29, 32–35, 37, 38, 40, 52] and in most cases, higher Visual Analogue Scale (VAS) global scores [14, 17, 21, 22, 29, 33, 35, 37, 38, 40] compared with those without FM. Studies reported conflicting results regarding the number of swollen joints in patients with comorbid FM. Swollen joint counts were significantly higher in RA complicated by FM in only 3 out of 15 reporting studies [17, 24, 25]. The remaining studies showed non-significant differences. There was a similarly stark contrast with ESR, which was not found to be statistically different between the comparison groups in any of the 15 studies in which it was reported.

**Fig. 2:**
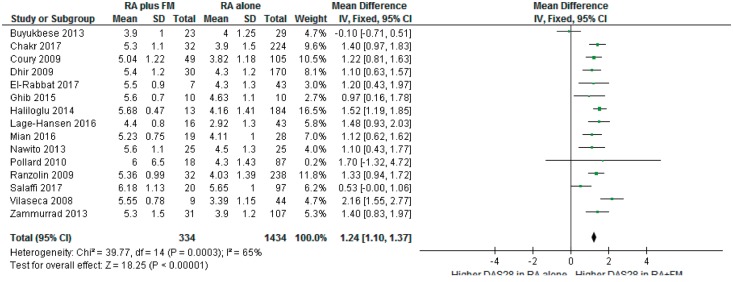
Fixed-effects meta-analysis of DAS28 in RA *vs* RA with comorbid FM df: degrees of freedom; iv: inverse variance.

Four studies reported disease outcomes as medians and ranges, suggesting a skewed distribution in those populations and, as such, precluding the possibility of including them in meta-analysis across all RA studies [14, 17, 21, 32]. For the remaining 15 studies it was possible to pool results in meta-analysis (see Fig. 2). Participants with FM and RA were found to have significantly higher pooled disease severity scores than those with RA alone (DAS28 mean difference 1.24; 95% CI: 1.10, 1.37). The level of heterogeneity was moderate in this case (*I*^2^ = 65%), but would have been much lower (*I*^2^= 37%) if it had not been for an outlying result reported by Buyukbese *et al.* [20]. On examination of this study it was observed that an older version of DAS rather than DAS28 was used for the composite assessment of disease activity, one which was later modified to DAS28 [54], and this could account for the discrepancy. After composite data from this study were excluded, the DAS28 mean difference was 1.30 (95% CI: 1.17, 1.44).

### AS and axial spondyloarthritis

Every paper reporting the prevalence of FM in AS adopted a cross-sectional study design [11, 13, 45, 47–50]. The prevalence of concomitant FM ranged from 4.11 to 25% and in proportional meta-analysis overall prevalence of FM was 13% (95% CI: 7, 19%) across all studies (see supplementary Fig. S2, available at *Rheumatology* online). Once again, heterogeneity was high between study estimates (*I*^2^ = 93.9%).

As above, in addition to evaluating the prevalence of FM in AS, some studies also investigated the effect of FM on disease activity. This was consistently assessed using BASDAI scores. The effect of FM on DAS in AS patients was reported by five studies [11, 13, 47–49]. Higher BASDAI scores were noted in all studies for participants with FM. This was found to be a statistically significant difference in all but one study, which did not report *P*-values [50]. As with RA, these studies generally did not report statistically significant differences for ESR or CRP levels in patients with and without FM.

**Fig. 3:**
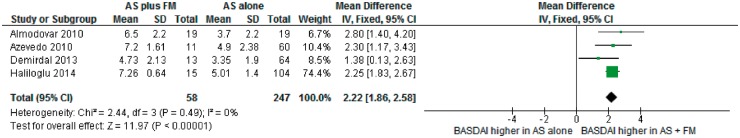
Fixed-effects meta-analysis of BASDAI in AS *vs* AS with comorbid FM df: degrees of freedom; iv: inverse variance.

One study did not report the full data for disease activity between comparison groups [47]. Otherwise, the consistent reporting of disease severity and the use of BASDAI across the remaining AS studies provided the opportunity to summarize results in meta-analysis (see Fig. 3). Participants with FM and AS were found to have significantly higher pooled DAS than those with AS alone (BASDAI mean difference 2.22; 95% CI: 1.86, 2.58). Heterogeneity was low (*I*^2^ = 0%), suggesting that measurements were consistent between studies with few important differences between the populations.

Additionally, four papers reported the prevalence of FM in axSpA populations defined using ASAS axSpA non-radiographic criteria [27], or radiological and non-radiological criteria together [45, 46, 50]; these all adopted a cross-sectional study design. The prevalence range of concomitant FM in these studies was 9.5–25.2%. Proportional meta-analysis across all axSpA groups (including AS) was not attempted due to fundamental differences in the classifications of the index disease groups.

Three studies reported the impact of comorbid FM on disease activity in axSpa [45, 46, 50]. These studies included both radiographic and non-radiographic defined axSpA. BASDAI scores were reported higher in all three studies among those with comorbid FM. In one study this was a statistically significant difference [45]. In another study, it was unclear if the difference was important since significance testing was not reported between those with and without FM [50]. Finally, Wach *et al.* [46] reported non-significant differences in BASDAI scores for participants with comorbid FM in axSpA.

### PsA

Six papers assessed the prevalence of FM in PsA, these all adopted a cross-sectional study design [27, 42–44, 50, 51]. The reported prevalence of concomitant FM in PsA ranged from 9.6 to 27.2%. In proportional meta-analysis, the overall prevalence of FM was 18% (95% CI: 13, 23%) across all studies (see supplementary Fig. S3, available at *Rheumatology* online). Heterogeneity was moderate between study estimates (*I*^2^ = 73.1%).

Two articles reported the effect of comorbid FM on disease activity [43, 51]. One study reported significantly higher DAS for the Composite Psoriatic Disease Activity Index (CPDAI), Minimal Disease Activity (MDA), Disease Activity Index for Psoriatic Arthritis (DAPSA), DAS28, BASDAI, and the Leeds Enthesitis Index (LEI) in participants with comorbid FM. As in other inflammatory conditions, no statistically significant effect was found between those with and without FM for swollen joint count or CRP [51]. In another study, the absence of comorbid FM was associated with a significantly increased rate of clinically diagnosed remission, defined as a documented absence of clinical signs: no tender joints, swollen joints, enthesitis or dactylitis (hazard ratio = 11.71; 95% CI: 1.61, 85.22) [43].

## Discussion

This review found that concomitant FM is common in chronic inflammatory arthritis. We found overall prevalence of FM to be 21% in RA (range 4.9–52.4%), 13% in AS (range 4.11–25.2%) and 18% in PsA (range 9.6–27.2%). Differences between diseases likely reflect the differing proportions of gender found naturally for each condition. RA affects more women [55], PsA occurs in men and women almost equally [56] and AS is found predominantly in men [57]. FM is strongly associated with female gender [4] and as such corresponds to the relative prevalence estimates found in these inflammatory disorders.

Heterogeneity was high in meta-analysis of concomitant FM prevalence, which suggests pooled results should be interpreted with caution. We stratified the meta-analysis by developed/non-developed populations, study sample selection methods and study risk of bias, but none of these factors had a significant impact on the amount of unexplained variability (data not shown). While prevalence estimates are also affected by the classification system used [7], we mitigated this effect in design by restricting to the ACR 1990 or 2010 classification of FM, and restricting to validated classification systems for the index conditions. Therefore, the variability in estimates is likely accounted for by the fact that studies differed by sample size, age and gender mix, all factors which are known to affect prevalence of FM [6]. Regardless of this variability, almost all individual studies reported rates of FM that were significantly higher than those reported for the general population (∼1–5%) [6, 7].

A greater consistency was found between studies reporting the impact of FM upon DAS. Included studies described an association between the presence of comorbid FM and worse DAS. A mean DAS28 difference of 1.24 was found in RA (95% CI: 1.10, 1.37) and a mean BASDAI difference of 2.2 was found in AS (95% CI: 1.86, 2.58). The estimated effect sizes were clinically important since a difference of 1–2 points in these disease scores could be the difference between starting or stopping potentially harmful, and expensive, biologic drugs. In PsA, the evidence was scarce, though the presence of comorbid FM was related to significantly higher DAS and decreased likelihood of clinical remission in reporting studies [43, 51].

This difference remained in people with otherwise similar demographic and index disease characteristics. For example, in the five studies that included only female participants, disease scores remained significantly higher in those with concomitant FM [21, 24, 29, 35, 40]. Additionally, other confounders such as age and disease duration were found to be broadly similar between groups across the majority of included studies. Therefore, it may be the presence of FM itself that inflates DAS.

Many authors attributed the observed influence of FM to the subjective, self-reported components that comprise significant portions of disease scoring in chronic inflammatory arthritis. For example, across RA studies, the higher total DAS was largely found to be due to tender joint count and VAS global scores, rather than due to elevation of more objective measures such as inflammatory markers (ESR, CRP) or swollen joint counts [14, 17, 21, 22, 24–26, 29, 32–35, 37, 38, 40]. Likewise in AS populations, studies concluded that there was no correlation between the DAS and severity of the physical findings [47], implying that the concomitant FM may have been responsible for the elevated BASDAI results [11, 13, 47–49]. Finally, in PsA, comorbid FM was found to have a negligible impact for objective measures such as swollen joint count or CRP [51].

We reviewed different DAS to compare for resilience to the effect of comorbid FM in RA. Similarly to DAS28, the simplified disease activity score [21] and the clinical disease activity score [21, 29, 35] were found to be amplified in the presence of FM. However, one study showed that US scores could be used to help distinguish between raised disease activity due to inflammation, and raised disease activity due to comorbid FM [21]. In PsA, comorbid FM was associated with significantly higher DAS for multiple disease scores including CPDAI, MDA, DAPSA, DAS28, BASDAI and LEI [51]. No studies in AS reported multiple disease scores for comparison.

This review has some limitations. The search was limited to English language papers and full text papers so that the content of the articles could be fully understood and assessed for quality. Included studies were mostly cross-sectional in design which, while enabling the investigation of several associations simultaneously, did not make it possible to infer causality. In studies that compared DAS, there was rarely adjustment or stratification for other important confounding factors. Age, gender and number of mental health conditions were shown to be different between groups with and without FM in various included publications. Presence of FM may also act as a proxy variable for underlying mental health problems since the presence of mental health disorders contributes to the classification of FM by ACR criteria [58]. Regardless, the reported impact associated with comorbid FM was found to be remarkably consistent across numerous studies in chronic inflammatory arthritis.

## Conclusion

This systematic review of 40 papers found that comorbid FM is much more common in patients with RA, AxSpA or PsA than in the general population. FM was significantly associated with higher DAS but not with higher objective clinical (swollen joint count) or laboratory (ESR, CRP) markers of disease activity. This review highlights the limitations of using disease activity indices alone in assessing inflammatory activity in rheumatic patients with concomitant FM. It is therefore important that these scores are interpreted in conjunction with knowledge of the presence of concomitant FM to ensure optimal management and appropriate drug treatment.

## Supplementary Material

Supplementary Data
