# Supplementary material for: Concomitant fibromyalgia complicating chronic inflammatory arthritis: a systematic review and meta-analysis
Source: Rheumatology (Oxford). 2018 May 16;57(8):1453–60. doi: 10.1093/rheumatology/key112 (PMC6055651; doi:10.1093/rheumatology/key112)
Supplement: Supplementary Data [file key112_suppl_data.docx]

SUPPLEMENTARY DATA

**Supplementary Table S1: Quality assessment scoring for all included cross-sectional studies using NOS criteria**

|  | **Sample representive** | **Sample size justified** | **Non-respondents described** | **Ascertainment of exposure** | **Comparability of groups** | **Outcome assessment** | **statistical test appropriate** | **TOTAL/ 10** | **TOTAL/5** |
| --- | --- | --- | --- | --- | --- | --- | --- | --- | --- |
| Almodovar 2010 (1) | * | 0 | 0 | ** | * | * | * | 6 | NA |
| Amity 2016 (2) | * | 0 | 0 | ** | NA | NA | NA | NA | 3 |
| Abbasi 2014 (3) | * | 0 | 0 | ** | ** | ** | * | 7 | NA |
| Ahmad 2015 (4) | 0 | 0 | 0 | ** | NA | NA | NA | NA | 2 |
| Aloush 2007 (5) | * | 0 | 0 | ** | 0 | * | 0 | 4 | NA |
| Brikman 2016 (6) | * | 0 | 0 | ** | * | ** | * | 7 | NA |
| Azevedo 2010 (7) | 0 | 0 | 0 | ** | 0 | ** | * | 5 | NA |
| Buyukbese 2013 (8) | 0 | 0 | 0 | ** | * | * | 0 | 4 | NA |
| Chakr 2016 (9) | * | * | 0 | ** | * | ** | * | 8 | NA |
| Christensen 2016 (10) | * | * | 0 | ** | NA | NA | NA | NA | 4 |
| Coury 2009 (11) | * | 0 | 0 | ** | * | ** | * | 7 | NA |
| Demirdal 2013 (12) | 0 | 0 | 0 | ** | * | * | 0 | 4 | NA |
| Di Carlo 2017 (13) | * | 0 | 0 | ** | NA | NA | NA | NA | 3 |
| Dhir 2009 (14) | * | 0 | 0 | ** | ** | * | * | 7 | NA |
| El-Rabbat 2017 (15) | 0 | 0 | 0 | ** | ** | * | * | 6 | NA |
| Fan 2017 (16) | * | 0 | 0 | ** | NA | NA | NA | NA | 3 |
| Gheita 2016 (17) | * | 0 | 0 | ** | NA | NA | NA | NA | 3 |
| Gist 2017 (18) | * | * | 0 | ** | NA | NA | NA | NA | 4 |
| Graceffe 2014 (19) | 0 | 0 | 0 | ** | 0 | ** | 0 | 4 | NA |
| Haliloglu 2014 (20) | * | 0 | 0 | ** | 0 | * | * | 5 | NA |
| Husted 2010 (21) | * | 0 | 0 | ** | NA | NA | NA | NA | 3 |
| Joharatnam 2015 (22) | * | 0 | 0 | ** | * | ** | * | 7 | NA |
| kapoor 2011 (23) | * | 0 | 0 | ** | ** | * | * | 7 | NA |
| Karpouzas 2012 (24) | * | 0 | 0 | ** | NA | NA | NA | NA | 3 |
| Lage-Hansen 2016 (25) | * | * | 0 | ** | ** | ** | 0 | 8 | NA |
| Lee 2017 (26) | 0 | 0 | 0 | ** | NA | NA | NA | NA | 2 |
| Macfarlane 2017 (27) | * | 0 | 0 | ** | NA | NA | NA | NA | 3 |
| Mian 2016 (28) | 0 | 0 | 0 | ** | 0 | * | 0 | 3 | NA |
| Pollard 2010 (29) | * | 0 | 0 | ** | 0 | 0 | * | 4 | NA |
| Ranzolin 2009 (30) | * | * | 0 | ** | * | ** | * | 8 | NA |
| Salaffi 2014 (31) | * | 0 | 0 | ** | 0 | * | * | 5 | NA |
| Salaffi 2017 (32) | 0 | 0 | 0 | ** | * | * | * | 5 | NA |
| Ton 2017 (33) | * | 0 | * | ** | 0 | * | * | 6 | NA |
| Urrows 1994 (34) | * | 0 | 0 | ** | NA | NA | NA | NA | 3 |
| Vilaseca 2008 (35) | * | 0 | 0 | ** | * | * | 0 | 6 | NA |
| Wach 2017 (36) | * | 0 | 0 | ** | * | * | * | 6 | NA |
| Zammurrand 2013 (37) | 0 | * | 0 | ** | ** | * | 0 | 6 | NA |

**Supplementary Table S2: Quality assessment scoring for all included case-control studies using NOS criteria**

|  | **Case definition** | **Representativeness cases** | **control selection** | **control definition** | **comparability** | **exposure ascertainment** | **same method case/control** | **non-response** | **TOTAL/10** |
| --- | --- | --- | --- | --- | --- | --- | --- | --- | --- |
| Chakr 2015 (38) | * | * | * | * | ** | * | * | 0 | 8 |
| Nawito 2013 (39) | * | * | * | 0 | ** | 0 | * | * | 7 |
| Ghib 2015 (40) | * | * | * | * | ** | * | * | 0 | 8 |

**Supplementary Table S3: Rheumatoid Arthritis Studies**

| Reference | Design | Criteria Used for Diagnosis of RA | Number of Study Patients with RA | Age, years, median (IQR)/ mean (SD)/ mean | Females in the Study, n (%) | Disease Duration, years, median (IQR)/ mean (SD)/ mean | Patients with RA and FM in the Study, n (%) | RA Patients with FM who are Female, n (%) | Disease Activity Score Used | DAS, median (IQR)/mean (SD) |
| --- | --- | --- | --- | --- | --- | --- | --- | --- | --- | --- |
| Abbassi L et al.  Fibromyalgia Complicating Disease Management in Rheumatoid Arthritis (2014) (3) | CS | Adapted ACR 1987 revised criteria | n=120 | 40 (32 -51) | n=110 (91.7) | Not Stated | n= (25.8) | n=31 (100) | DAS28 | Not Stated |
| Ahmad et al.  Association of antithyroid peroxidase antibody with fibromyalgia in rheumatoid arthritis.(2015) (4) | CS | ACR 1987 revised criteria | n= 204 | 58.23 (13.06) | 184 (92) | Not stated | 51 (25)- only amongst those with an indication for TFTs | Not stated | Not used | N/A |
| Amity et al  Agreement of Physicians and Nurses Performing Tender and Swollen Joint Counts in Rheumatoid Arthritis (2016) (2) | CS | 2010 ACR/EULAR criteria | N= 72 | 62.1 (11.4) | N=54 (75) | 17.8 (13.3) | N= 26 (36.6) | Not stated | Not used | N/A |
| Buyukbese et al,  Effect of fibromyalgia on bone mineral density in patients with fibromylagia and rheumatoid arthritis (2013) (8) | CS | ACR 1987 revised criteria | N= 52 | 53 (9.9) | Unclear how many RA participants were females | Not stated for whole RA group | N= 23 (44.2) | Not stated | DAS | Not stated for whole RA group |
| Chakr et al  Is Ultrasound a Better Target than Clinical Disease Activity Scores in Rheumatoid Arthritis with Fibromyalgia? A Case-Control Study (2015) (38) | Case control | ACR/ European League Against Rheumatism criteria 2010 | N=72 | 57.5 (49.3–66.8) | 72 (100) | Not stated for whole RA group. | N=36 (selected cc) | N=36 (100) | DAS28 | Not stated for whole RA group |
| Chakr et al  Rheumatoid arthritis seems to have DMARD treatment decision influenced by fibromyalgia (2016) (9) | CS | ACR 1987 Criteria | N=256 | 55.4 (12.6) | 216 (84.4) | 11.1 (7.4) | N=32 (12.5) | 31 (96.9) | DAS28 | 4.1 (1.9) |
| Christensen et al  Non-nociceptive pain in rheumatoid arthritis is frequent and affects disease activity estimation: cross-sectional data from the FRAME study (2016) (10) | CS | ACR 1987 or 2010 Criteria | N= 102 | Not stated for whole cohort | 76 (74.5) | Not stated for whole cohort | N=38 (37.2) | Not stated | DAS28-P | Not stated for whole cohort |
| Coury et al.  Rheumatoid arthritis and fibromyalgia: a frequent unrelated association complicating disease management. (11) | CS | ACR 1987 revised criteria | N=154 | 57.7 (14.5) | All female | 15.5 (13.8) | N=49 (31.8) | N=49 (100) | DAS28-ESR | Not Stated |
| Dhir V et al.  Fibromyalgia is Common and Adversely Affects Pain and Fatigue Perception in North Indian Patients with Rheumatoid Arthritis (2009) (14) | CS | ACR 1987 Criteria | n=200 | 46.8 (10.7) | n=167  (83.5) | Not Stated | n=30  (15) | n=27 (90) | DAS28 with 3 variables  (DAS28-3v) | Not Stated |
| El-Rabbat et al  Clinical significance of fibromyalgia syndrome in different rheumatic diseases: Relation to disease activity and quality of life (2017) (15) | CS | ACR/ European League Against Rheumatism criteria 2010 | N=50 | Not stated for whole cohort | N=45 | Not stated for whole cohort | N=7 (16.3) | N=7 (100) | DAS28 | Not stated for whole cohort |
| Fan et al  Frequency of concomitant fibromyalgia in rheumatic diseases: Monocentric study of 691 patients.(2017) (16) | CS | ACR/ European League Against Rheumatism criteria 2010 | N=325 | Not stated for RA cohort | 236 (72.8) | Not stated for RA cohort | n= 16 (4.9) | Not Stated | Not Stated | Not Stated |
| Gheita et al  Vitamin D status in rheumatoid arthritis patients: relation to clinical manifestations, disease activity, quality of life and fibromyalgia syndrome (2016) (17) | CS | ACR/ European League Against Rheumatism criteria 2010 | N=63 | 41.59 (9.69) | 49 (77.7) | 5.89 (3.67) | N=33  (52.4) | Not stated | DAS28 | Not stated |
| Ghib et al  The role of ultrasonography in assessing disease activity in patients with rheumatoid arthritis and associated fibromyalgia (2015) (40) | Case Control | ACR/ European League Against Rheumatism criteria 2010 | N=20 | Not stated for whole cohort | N=20 (selected) | Not stated for whole cohort | N=10 (100) (selected) | N=10 (100) selected | DAS28 | Not stated for whole cohort |
| Gist et al  Fibromyalgia remains a significant burden in rheumatoid arthritis patients in Australia (2017) (18) | CS | ACR/ European League Against Rheumatism criteria 2010 | N=117 | 62.1 (12.6) | 88 (75.2) | Not stated | N=39 (33.3) | 40 (81.6) | Not stated | Not stated |
| Karpouzas et al  Correlates and Predictors of Disability in Vulnerable US Hispanics With Rheumatoid Arthritis (2012) (24) | CS | ACR 1987 revised criteria | N=251 | 51.5 (11) | 216 (86) | 10.2 (8.3) | 39 (16) | Not stated | DAS28-ESR | 3.3 (1.2) |
| Lee et al.  Pain Sensitization is Associated with Disease Activity in Rheumatoid Arthritis Patients: A Cross-Sectional Study (2017) (26) | CS | ACR/ European League Against Rheumatism criteria 2010 | N=139 | 54.2 (13.6) | 116 (83.5) | 9.3 (12.7) | 44 (31.7) | Not stated | CDAI | 24.4 (14.0) |
| Haliloglu et al.  Fibromyalgia in Patients with Other Rheumatic Diseases: Prevalence and Relationship with Disease Activity (2014) (20) | CS | ACR 1987 revised criteria | n=197 | Not Stated | n=158  (80.2) | Not Stated | n=13 (6.6) | n=13 (100) | DAS28 | Not Stated |
| Joharatnam N et al.  A Cross-Sectional Study of Pain Sensitivity, Disease-Activity Assessment, Mental Health, and Fibromyalgia Status in Rheumatoid Arthritis (2015) (22) | CS | ACR/ European League Against Rheumatism criteria 2010 | n=50 | 60 (54-69) | n=38 (76) | Not Stated | n=24 (48) | n=18 (75) | DAS28, DAS28-P | Not Stated |
| Kapoor SR et al.  Fibromyalgia in Patients with Rheumatoid Arthritis: Driven by Depression or Joint Damage? (2011) (23) | CS | ACR 1987 revised criteria | n=285 | 59.5 (10.2) | n=202 (71) | 10.9 (9.3) | n=18 (6.3) | n=16 (89) | DAS28 | Not Stated |
| Lage-Hansen et al  Concomitant fibromyalgia in rheumatoid arthritis is associated with the more frequent use of biological therapy: a cross-sectional study (2016) (25) | CS | ACR 1987 revised criteria or ACR/ European League Against Rheumatism criteria 2010 | N= 162 | Not stated for whole cohort | N=121 (74.7) | Not stated for whole cohort | N= 25 (15.4) | 23 (92) | DAS28 | Not stated for whole cohort |
| Mian et al  Rheumatoid arthritis patients with  fibromyalgic clinical features have  significantly less synovitis as defined by  power Doppler ultrasound (2016) (28) | CS | ACR 1987 revised criteria or ACR/ European League Against Rheumatism criteria 2010 | N=47 | Mean 61 years | 38 (81) | Not stated | N= 19 (40.4) | 16 (84) | DAS28 | Mean 4.5 |
| Nawito et al,  The impact of fibromyalgia on disease assessment in rheumatoid arthritis patients (2013) (39) | Case control | ACR/ European League Against Rheumatism criteria 2010 | N=50 | Not stated for whole cohort | 50 (100) | Not stated for whole cohort | N= 25 (50% selected) | Not stated | DAS28 | Not stated for whole cohort |
| Pollard et al.  Fibromyalgic rheumatoid arthritis and disease assessment.(2010) (41)  Pain Thresholds in Rheumatoid Arthritis: The Effect of Tender Point Counts and Disease Duration (2012) (29) | CS | ACR criteria, unreferenced | n=105 | Mean 60 years (range 24–88 years); | 80 (76.2) | 13 years (range  0.1–54), | n=18 (17) | Not Stated | DAS28 | Not Stated |
|  | CS | ACR criteria, unreferenced | n=100 | Mean 62 years  (range 25–86 years) | 77 (77) | Not stated | n=12 (12) | Not Stated | DAS28 | Not Stated |
| Ranzolin A et al.  Association of Concomitant Fibromyalgia with Worse Disease Activity Score in 28 Joints, Health Assessment Questionnaire, and Short Form 36 Scores in Patients with Rheumatoid Arthritis (2009) (30) | CS | ACR 1987 revised criteria | n=270 | Not Stated | n=228 (84.4) | Not Stated | n=32 (11.9) | n=31 (96.9) | DAS28 | Not Stated |
| Salaffi et al.  The influence of fibromyalgia on achieving remission in patients with long-standing rheumatoid arthritis (2017) (32) | Data CS from baseline (study longitudinal) | ACR/ European League Against Rheumatism criteria 2010 | n=117 | 58.5 (11.7) | 90 (77.2) | 11.2 (8.7) | N=20 (17.1) | Not stated | CDAI  SDAI | 31.9 (9.4)  33.4 (10.2) |
| Ton et al  Look Beyond the Disease Activity Score of 28 Joints (DAS28): Tender Points Influence the DAS28 in Patients with Rheumatoid Arthritis (2012) (33) | CS | ACR 1987 revised criteria | N=196 | 59 (40–78) median 10^th^ to 90^th^ percentile | 137 (70) | 4 (1–8) median 10^th^ to 90^th^ percentile | N=29 (14.7) | 24 (83) | DAS28 | 3.1 (1.3–5.1) median 10^th^ to 90^th^ percentile |
| Urrows S et al.  Unique Clinical and Psychological Correlates of Fibromyalgia Tender Points and Joint Tenderness in Rheumatoid Arthritis (1994) (34) | CS | ACR 1958 revised criteria | n=67 | 52.6 (12.4) | n=49 (73) | 8.19 (6.98) | n=5 (7.5) | Not Stated | Not Used | N/A |
| Vilaseca et al  Effect of the Coexistence of Fibromyalgia in the DAS28 Index in Women With Rheumatoid Arthritis (2008) (35) | CS | ACR 1987 revised criteria | N=65 | Not stated for whole cohort | N=65 (100) | Not stated for whole cohort | 9 (17) | 9 (100) | DAS28 | Not stated for whole cohort |
| Zammurrad et al  Disease Activity Score in Rheumatoid Arthritis with or without Secondary Fibromyalgia (2013) (37) | CS | ACR/ European League Against Rheumatism criteria 2010 | N=138 | 42.9 (12.0) | 127 (92) | Not stated for whole cohort | 31 (22.5) | 30 (96.8) | DAS 28 | Not stated for whole cohort |

CS: Cross-sectional; DAS28-P: the proportion of DAS28 derived from the patient-reported components of visual analogue score and tender joint count), or fibromyalgia status.

**Supplementary Table S4: Axial and Ankylosing Spondylitis Studies**

| Reference | Design | Criteria Used for Diagnosis of AS | Number of Study Patients with AS | Age, years, median (IQR)/ mean (SD)/ mean | Females in the Study, n (%) | Disease Duration, years, median (IQR)/mean (SD)/mean | *n* (%) with AS and FM in the Study | *n* (%) of AS Patients with FM who are Female | Disease Activity Score Used | DAS, median (IQR)/mean (SD) |
| --- | --- | --- | --- | --- | --- | --- | --- | --- | --- | --- |
| **Ankylosing Spondylitis** | | | | | | | | | | |
| Almodóvar R et al.  Fibromyalgia in Patients with Ankylosing Spondylitis: Prevalence and Utility of the Measures of Activity, Function and Radiological Damage (2010) (1) | CS | Modified New York Criteria for AS | n=462 | 50 (12) | 120 (26) | Not Stated | n=19 (4.11) | n=13 (68) | BASDAI | Not Stated |
| Aloush V et al.  Fibromyalgia in Women with Ankylosing Spondylitis (2007) (5) | CS | Modified New York Criteria for AS | n=36 | Men: 40.3  Women: 48.7 | n=18 (50) | Men: 12.5  Women: 17.2 | n=9 (25) | n=9 (100) | BASDAI | Not Stated |
| Azevedo VF et al.  Occurrence of Fibromyalgia in Patients with Ankylosing Spondylitis (2010) (7) | CS | Modified New York Criteria for AS | n=71 | 43.67 (11.78) | n=17 (15.5) | 16.59 (9.64) | n=11 (15) | n=6 (54.5) | BASDAI | Not Stated |
| Demirdal et al  Coexisting of fibromyalgia syndrome and ankylosing spondylitis (2013) (12) | CS | Modified New York Criteria for AS | N= 77 | 37.96 (12.17) | N= 16 (20.8 ) | 10.4 (8.9) | n=13 (16.9) | N=5 (38.5) | BASDAI | 3.59 (1.99) |
| Fan et al  Frequency of concomitant fibromyalgia in rheumatic diseases: Monocentric study of 691 patients.(2017) (16) | CS | New York criteria for AS | N=137 | Not stated for AS cohort | Not stated for AS cohort | Not stated for AS cohort | n= 9 (6.4) | Not Stated | Not Stated | Not Stated |
| Haliloglu et al.  Fibromyalgia in Patients with Other Rheumatic Diseases: Prevalence and Relationship with Disease Activity (2014) (20) | CS | Bennet & Wood  Population studies of rheumatic diseases 1966 | n=119 | Not Stated | n=58 (48.7) | Not Stated | n=15 (12.6) | n=14 (93.3) | BASDAI | Not Stated |
| Salaffi F et al.  Fibromyalgia in Patients with Axial Spondylo-arthritis: Epidemiological Profile and Effect on Measures of Disease Activity (2014) (31) | CS | Modified New York Criteria for AS | Part A: n=211 | 52 (11) | n=51 (24.2) | Men: 7.1  Women: 7.8 | n=27 (12.7) | Not Stated | Versions of the ASDAS and BASDAI | Not stated for this subset |
| Macfarlane et al  The co-occurrence and characteristics of patients with axial spondyloarthritis who meet criteria for fibromyalgia: results from a UK national register (BSRBR-AS) (2017) (27) | CS | Modified New York Criteria for AS | N=1026 | Not Stated for this subset | Not Stated for this subset | Not Stated for this subset | N= 202 (19.7) | Not Stated for this subset | BASDAI | Not Stated for this subset |
| **Other Axial SpA** | | | | | | | | | | |
| Fan et al  Frequency of concomitant fibromyalgia in rheumatic diseases: Monocentric study of 691 patients.(2017) (16) | CS | ASAS criteria for non- radiographic SpA, | N=64 | Not stated for nr-axSpA cohort | Not stated for nr SpA cohort | Not stated for nr SpA cohort | n= 15 (23.9) | Not Stated | Not Stated | Not Stated |
| Macfarlane et al  The co-occurrence and characteristics of patients with axial spondyloarthritis who meet criteria for fibromyalgia: results from a UK national register (BSRBR-AS) (2017) (27) | CS | ASAS imaging  criteria but not mNY criteria for AS | N=399 | Not Stated for this subset | Not Stated for this subset | Not Stated for this subset | N= 101 (25.2) | Not Stated for this subset | BASDAI | Not Stated for this subset |
|  |  | ASAS non-radiological criteria | N= 65 | Not Stated for this subset | Not Stated for this subset | Not Stated for this subset | N= 6  (9.5) | Not Stated for this subset | BASDAI | Not Stated for this subset |
|  |  | axSpA meeting New York criteria for AS or either ASAS criteria for axSpA | N=1504 | 51.2 (40.1-63.1) | 479 (31.8) | 19 (9-33) | N= 311 (20.7) | Not Stated for this subset | BASDAI | Not Stated for this subset |
| Salaffi F et al.  Fibromyalgia in Patients with Axial Spondyloarthritis: Epidemiological Profile and Effect on Measures of Disease Activity (2014) (31) | CS | Definite axial-SpA: all meet Modified New York Criteria for AS and ASAS criteria for axial SpA. (AS or axial PsA) | N=402 | 52 (11) | N=127 (31.6) | Men: 7.1  Women: 7.8 | n=59 (14.9) | Not Stated | BASDAI  ASDAS | Total BASDAI: 4.5 (3.6-5.6)/ 4.6 (1.3) |
| Wach et al  Fibromyalgia in Spondyloarthritis: Effect on Disease Activity Assessment in Clinical Practice (36) | CS | ASAS classification criteria for axSpA | n=81 | Not stated for whole cohort | 24 (29.6) | Not stated for whole cohort | n=12 (14.8) | n= 6 (50.0) | BASDAI | Not stated for whole cohort |

ASDAS: Ankylosing Spondylitis Disease Activity Index; ASAS: Assessment in Spondyloarthritis International Society; AxSpA: Axial Spondyloarthritis (includes AS and psoriatic arthritis patients)

**Supplementary Table S5: Peripheral and Psoriatic Arthritis**

|  |  |  |  |  |  |  |  |  |  |  |  |
| --- | --- | --- | --- | --- | --- | --- | --- | --- | --- | --- | --- |
| Reference | Design | Criteria Used for Diagnosis of pSpA or PsA | Study Patients with AxSpA or PsA, N | Age, years, median (IQR)/ mean (SD)/ mean | Females in the Study, n (%) | Disease Duration, years (unless stated), median (IQR)/ mean (SD)/ Mean | Patients pSpA or PsA *and* FM in the Study, n (%) | pSpA or PsA Patients with FM who are Female, n (%) | DAS Used | DAS, median (IQR)/ mean (SD) (whole cohort) |  |
| **Psoriatic arthritis** | | | | | | | | | | | |
| Brikman et al  The Effect of the Presence of Fibromyalgia on Common Clinical Disease Activity Indices in Patients with Psoriatic Arthritis: A Cross-sectional Study (2016) (6) | CS | CASPAR | N=73 | 51.7 (13.5) | 42 (57.5) | 10.3 (7.5) | 13 (17.8) | 12 (92.3) | CPDAI  MDA  DAPSA  DAS28  PASI | Not stated |  |
| Di Carlo et al  The 12-item Psoriatic Arthritis Impact of Disease Questionnaire: Construct Validity, Reliability, and Interpretability in a Clinical Setting (2017) (13) | CS | CASPAR | N=144 | 51.4 (12.8) | 63  (43.8) | 10.3 (8.0) | 27 (18.8) | Not stated | DAPSA  cDAPSA | Not stated |  |
| Fan et al  Frequency of concomitant fibromyalgia in rheumatic diseases: Monocentric study of 691 patients (2017). (16) | CS | CASPAR | N=59 | Not stated for PsA cohort | Not stated for PsA cohort | Not stated for PsA cohort | n= 6 (9.6) | Not Stated | Not Stated | Not Stated |  |
| Graceffa et al  Clinical remission of psoriatic arthritis in patients receiving continuous biological therapies for 1 year: the experience of an outpatient dermatological clinic for psoriasis (2015) (19) | CS | CASPAR | N=74 | 48 (16) | 26 (35.1) | 3 (6) | 12 (16) | Not Stated | Clinical remission | Month 3= 23 (31%)  Month 6= 33 (44.6%)  Month 12 = 36 (48.6%) |  |
| Husted et al  Longitudinal Analysis of Fatigue in Psoriatic Arthritis (2010) (21) | CS | Classification of Psoriatic Arthritis study group criteria | N=390 | 48.3 (12.3) | 159 (40.8) | 12.9 (9.9) | 106 (27.2) | Not Stated | PASI | 3 (0.9, 6.45) |  |
| Salaffi F et al.  Fibromyalgia in Patients with Axial Spondylo-arthritis: Epidemiological Profile and Effect on Measures of Disease Activity (2014) (31) | CS | ASAS criteria for axial-SpA with origin of PsA | N=191 | Not stated for this subset | N=76 (39.8) | Men: 7.1  Women: 7.8 | 33 (17.2) | Not stated for this subset | Part A: Not Used  Part B: Different Versions of the ASDAS and BASDAI | Part A: N/A  Part B: Total BASDAI – 4.5 (3.6-5.6)/ 4.6 (1.3) |  |

CASPAR: Classification Criteria for Psoriatic Arthritis; CPDAI: Composite Psoriatic Disease Activity Index; MDA: Minimal Disease Activity; DAPSA: Disease Activity Index for Psoriatic Arthritis; DAS28: Disease Activity Score of 28 joints; PASI: Psoriasis Activity and Severity Index.

**Supplementary Table S6: Comparison of DAS between patients with and without FM in the RA studies**

| Reference | | Number of Patients | Age, years, median (IQR)/ mean (SD) | Females, n (%) | Disease Duration, years, median (IQR)/ mean (SD) | Disease Activity Indices Used | Disease Activity Score | | | | |
| --- | --- | --- | --- | --- | --- | --- | --- | --- | --- | --- | --- |
|  |  |  |  |  |  |  | DAS, median (IQR)/mean (SD) | Total Number of Tender joints, 0-28, median (IQR)/mean (SD) | Number of Swollen Joints, 0-28, median (IQR)/mean (SD) | Visual Analogue Scale, mm, median (IQR)/ mean (SD) | ESR, mm/hr, median (IQR)/ mean (SD) |
| Abbassi L et al.  Fibromyalgia Complicating Disease Management in Rheumatoid Arthritis (2014) (3) | RA only | n=89 | 40 (30-52) | n=79 (88.8) | Not Stated | DAS28 Score | 4.9 (3.66-5.71) | 5 (2-12.5) | 3 (1-7) | 20 (10-30) | 40 (22-60) |
|  | RA & FM | n=31 | 38 (35-45) | n=31 (100) | Not Stated | DAS28 Score | 7.04 (6.62-7.64) | 24 (24-28) | 9 (2-18) | 80 (70-80) | 37 (26-48) |
| Buyukbese et al,  Effect of fibromyalgia on bone mineral density in patients with fibromylagia and rheumatoid arthritis (2013) (8) | RA only | N= 29 | 52.7 (8.2) | Not stated | 7.65 (6.9) | DAS | 4 (1.25) | Not stated | Not Stated | Not stated | Not Stated |
|  | RA & FM | N= 23 | 53.5 (12) | Not stated | 7.7 (6.5) | DAS | 3.9 (1) | Not stated | Not stated | Not stated | Not stated |
| Coury et al.  Rheumatoid arthritis and fibromyalgia: a frequent unrelated association complicating disease management. (2009) (11) | RA only | n=105 | 57.7 (14.5) | n=105 (100) | 15.5 (13.8) | DAS28-ESR | 3.82 (1.18) | 4.8 (6.05) | 3.22 (3.64) | Not Stated | 28.52 (22.63) |
|  | RA & FM | n=49 | 60.9 (12.3) | n=49 (100) | 12.8 (9) | DAS28-ESR | 5.04 (1.22) | 15.04 (9.67) | 5.02 (6.62) | Not Stated | 32.67 (23.44) |
| Chakr et al  Is Ultrasound a Better Target than Clinical Disease Activity Scores in Rheumatoid Arthritis with Fibromyalgia? A Case-Control Study (2015) (38) | RA only | N= 36 | 57.5 (48.3–66.8) | N= 36 (100) | 13.0 (6.0, 17.8) | DAS28 | 4.0 range- 3.3, 4.6 | 4.0 (2.0–6.0) | 3.0 range- 2.0, 6.0 | 38.0 range 17.3-59.3 | 19.5 (9.0-32.8) |
|  |  |  |  |  |  | SDAI | 13.1 (9, 22.4) |  |  |  |  |
|  |  |  |  |  |  | CDAI | 13.1 (8, 22.1) |  |  |  |  |
|  |  |  |  |  |  | GS-US7 score | 9 (7, 11) |  |  |  |  |
|  |  |  |  |  |  | PD-US7 score | 4 (2, 5) |  |  |  |  |
|  | RA & FM | N= 36 | 57.5 (50.0, 66.5) | N= 36 (100) | 12.5 (6.0, 19.0) | DAS28 | 5.2 range- 4.3, 6.3 | 14.0 (12.0–16.5) | 5.0 range- 1.3, 8.0 | 59.5 range 39.5- 76.8 | 12.5 (7.0, 31.0) |
|  |  |  |  |  |  | SDAI | 31.1 (18, 40.3) |  |  |  |  |
|  |  |  |  |  |  | CDAI | 30.4 (18, 39.7) |  |  |  |  |
|  |  |  |  |  |  | GS-US7 score | 10 (7, 11) |  |  |  |  |
|  |  |  |  |  |  | PD-US7 score | 3 (1, 5.8) |  |  |  |  |
| Chakr et al  Rheumatoid arthritis seems to have DMARD treatment decision influenced by fibromyalgia (2016) (9) | RA only | N= 224 | 54.7 (12.5) | N= 185 (82.6) | 11.1 (7.3) | DAS28 | 3.9 (1.5) | 3.0 (0.0–8.0) | 2.4 (0.0–5.0) | 31.5 (14.0–52.2) | 26.0 (14.0–41.2) |
|  | RA & FM | N= 32 | 59.9 (12.8) | N= 31 (96.9) | 11.0 (7.7) | DAS28 | 5.3 (1.1) | 10.0 (5.0–17.0) | 3.9 (1.0–5.5) | 56.5 (41.5–90.0) | 28.5 (15.5–49.0) |
| Dhir V et al.  Fibromyalgia is Common and Adversely Affects Pain and Fatigue Perception in North Indian Patients with Rheumatoid Arthritis (2009) (14) | RA only | n=170 | 46.9 (10.3) | 140 (82.4) | 8.6 (6.1) | DAS28-3 | 4.3 (1.2) | 4.5 (5.7) | 2.2 (3.6) | Not Stated | 46.7 (25.3) |
|  | RA & FM | n=30 | 46.3 (12.4) | 27 (90) | 8.6 (6.6) | DAS28-3 | 5.4 (1.2) | 11.4 (8.9) | 4.3 (4.3 | Not Stated | 54.5 (26) |
| El-Rabbat et al  Clinical significance of fibromyalgia syndrome in different rheumatic diseases: Relation to disease activity and quality of life (2017) (15) | RA only | N=43 | 43.9 (11.8) | 38 (88.4) | 8.3 (6.4) | DAS28 | 4.3 (1.3) | Not Stated | Not Stated | Not Stated | 41.9(22.4) |
|  | RA & FM | N=7 | 45.1 (8.9) | 7 (100) | 8.7(8.4) | DAS28 | 5.5 (0.9) | Not Stated | Not Stated | Not Stated | 33.8 (11.1) |
| Ghib et al  The role of ultrasonography in assessing disease activity in patients with rheumatoid arthritis and associated fibromyalgia (2015) (40) | RA only | N=10 | 57.9 (14.8) | 10 (100) | 69 (55,135)- months | DAS28 | 4.63 (1.1) | 5.5 (2,10) | 6 (0.7,7.5) | 22 (13.7, 40) | 44.5 (21) |
|  |  |  |  |  |  | CDAI | 19.8 (9.7) |  |  |  |  |
|  |  |  |  |  |  | US GS score | 16(7.5, 24.7) |  |  |  |  |
|  |  |  |  |  |  | US PD score | 3.5(0, 10.7) |  |  |  |  |
|  | RA & FM | N=10 | 58 (7.1) | 10 (100) | 126 (71.5,168)- months | DAS28 | 5.6 (0.7) | 15 (11.7,20.7) | 5 (0.7,8) | 23 (7.2,42.5) | 66 (15) |
|  |  |  |  |  |  | CDAI | 30.9 (9.4) |  |  |  |  |
|  |  |  |  |  |  | US GS score | 9.5 (4.7, 13) |  |  |  |  |
|  |  |  |  |  |  | US PD score | 1.5(1, 8) |  |  |  |  |
| Haliloglu et al.  Fibromyalgia in Patients with Other Rheumatic Diseases: Prevalence and Relationship with Disease Activity (2014) (20) | RA only | n=184 | 53.09 (13.12) | 145 (78.8) | Not Stated | DAS28 | 4.16 (1.41) | Not Stated | Not Stated | Not Stated | 39.29 (28.22) |
|  | RA & FM | n=13 | 42.15 (8.25) | 13 (100) | Not Stated | DAS28 | 5.68 (0.47) | Not Stated | Not Stated | Not Stated | 30.72 (16.74) |
| Joharatnam N et al.  A Cross-Sectional Study of Pain Sensitivity, Disease-Activity Assessment, Mental Health, and Fibromyalgia Status in Rheumatoid Arthritis (2015) (22) | RA only | n=26 | 63 (54-70) | n=20 (77) | Not Stated | DAS28 | 4.4 (3.8-4.9) | 6 (4-9) | 1 (1-2) | 42 (24-55) | 17 (12-26) |
|  |  |  |  |  |  | DAS28-P | 0.50 (0.45-0.57) |  |  |  |  |
|  | RA & FM | n=24 | 58 (54-66) | n=18 (75) | Not Stated | DAS28 | 4.8 (4.4-5.3) | 11 (7-18) | 1 (0-2) | 70 (55-78) | 19 (8-29) |
|  |  |  |  |  |  | DAS28-P | 0.58 (0.52-0.64) |  |  |  |  |
| Kapoor SR et al.  Fibromyalgia in Patients with Rheumatoid Arthritis: Driven by Depression or Joint Damage? (2011) (23) | RA only | n=267 | 60 (53-67) | 186 (70) | 8 (3-16) | DAS28 | 4.11 (3.16-5.14) | 4 (1-8) | 3 (1-6) | 40 (21-59) | 18 (10-32) |
|  | RA & FM | n=18 | 62.5 (59-64) | 16 (89) | 13.5 (8-23) | DAS28 | 4.76 (4.11-5.34) | 8 (6-13) | 4 (1-6) | 47 (33-56) | 23 (15-54) |
| Lage-Hansen et al  Concomitant fibromyalgia in rheumatoid arthritis is associated with the more frequent use of biological therapy: a cross-sectional study (2016) (25) | RA tx with bio only | N= 43 | Not Stated | Not Stated | Not Stated | DAS28 | 2.92 (1.3) | 3.23 (4.9) | 0.91 (1.8) | 35.88 (25.1) | Not Stated |
|  | RA tx with bio & FM | N=16 | Not Stated | Not Stated | Not Stated | DAS28 | 4.40 (0.8) | 10.1 (7.5) | 2.25 (3.1) | 70.44 (17.8) | Not Stated |
| Mian et al  Rheumatoid arthritis patients with fibromyalgic clinical features have significantly less synovitis as defined by power Doppler ultrasound (2016) (28) | RA only | N= 28 | Mean 63 | 22 (79) | Not stated | DAS28 | 4.11 (1.00) | 7.39 (5.40) | 2.79 (3.51) | 49.50 (20.52) | 19.18 (17.84) |
|  | RA & FM | N=19 | Mean 57 | 16 (84) | Not stated | DAS28 | 5.23 (0.75) | 15.663 (5.84) | 1.84 (2.29) | 57.05 (18.31) | 20.53 (14.44) |
| Pollard et al.  Fibromyalgic rheumatoid arthritis and disease assessment. (2010) (41)  Pain Thresholds in Rheumatoid Arthritis: The Effect of Tender Point Counts and Disease Duration (2012) (33) | RA only | n=87 | Not Stated | Not Stated | Not Stated | DAS28 | 4.3 (1.43) | 6 (4.76) | 4 (0) | Not Stated | 27 (23.79) |
|  | RA & FM | n=18 | Not Stated | Not Stated | Not Stated | DAS28 | 6.0 (6.5) | 17 (8.66) | 4 (4.33) | Not Stated | 39 (34.63) |
| Ranzolin A et al.  Association of Concomitant Fibromyalgia with Worse Disease Activity Score in 28 Joints, Health Assessment Questionnaire, and Short Form 36 Scores in Patients with Rheumatoid Arthritis (2009) (30) | RA only | n=238 | 55.0 (12.4) | 197 (82.2) | Not Stated | DAS28 | 4.03 (1.39) | 3.0 (0.0-8.0) | 2.0 (0.0-5.0) | 32.0 (14.0-53.2) | 25.0 (13.7-40.0) |
|  | RA & FM | n=32 | 60.2 (12.8) | 31 (96.9) | Not Stated | DAS28 | 5.36 (0.99) | 9.5 (4.5-16.0) | 3.5 (1.0-5.0) | 56.5 (42.5-89.5) | 29.0 (16.0-49.0) |
| Salaffi et al.  The influence of fibromyalgia on achieving remission in patients with long-standing rheumatoid arthritis (2017) (32) | RA only | n=97 | 58.1 (12.1) | Not stated | 11.2 (8.5) | DAS28-CRP | 5.01 (0.86) | 9.47 (5.45) | 5.88 (4.18) | 7.03 (1.97) | 39.42 (23.65) |
|  |  |  |  |  |  | DAS28-ESR | 5.65 (1.00) |  |  |  |  |
|  |  |  |  |  |  | CDAI | 31.55 (9.17) |  |  |  |  |
|  |  |  |  |  |  | SDAI | 32.69 (9.44) |  |  |  |  |
|  |  |  |  |  |  | RADAI | 6.19 (1.57) |  |  |  |  |
|  |  |  |  |  |  | PRO-CLARA | 5.61 (1.98) |  |  |  |  |
|  | RA & FM | n= 20 | 60.0 (9.5) | Not stated | 11.3 (9.7) | DAS28-CRP | 5.37 (1.17) | 12.35 (7.11) | 7.05 (6.25) | 7.15 (1.95) | 41.60 (24.44) |
|  |  |  |  |  |  | DAS28-ESR | 6.18 (1.13) |  |  |  |  |
|  |  |  |  |  |  | CDAI | 33.30 (10.49) |  |  |  |  |
|  |  |  |  |  |  | SDAI | 36.97 (13.05) |  |  |  |  |
|  |  |  |  |  |  | RADAI | 6.90 (1.31) |  |  |  |  |
|  |  |  |  |  |  | PRO-CLARA | 6.25 (1.87) |  |  |  |  |
| Ton et al  Look Beyond the Disease Activity Score of 28 Joints (DAS28): Tender Points Influence the DAS28 in  Patients with Rheumatoid Arthritis (2012) (33) | RA only | n=167 | Not Stated | Not Stated | Not Stated | Not Stated | Not Stated | Not Stated | Not Stated | Not Stated | Not Stated |
|  | RA & FM | n=29 | 61 (43–79 median 10^th^ to 90^th^ percentile) | 24 (83) | 5 (1–8) median 10^th^ to 90^th^ percentile | DAS28 | 4.0 (1.2) | 5 (0–19) median 10^th^ to 90^th^ percentile | 1 (0–8) median 10^th^ to 90^th^ percentile | 42 (10–70) median 10^th^ to 90^th^ percentile | 15 (5–42) median 10^th^ to 90^th^ percentile |
| Nawito et al,  The impact of fibromyalgia on disease assessment in rheumatoid arthritis patients (2013) (39) | RA only | N= 25 | 45.3 (11.5) | 25 (100) | 7.5 (7.1) | DAS28 | 4.5 (1.3) | 4.5 (4.2) | 3 (4.1) | 46.8 (25.9) | 41.8 (22.5) |
|  |  |  |  |  |  | CDAI | 13.7 (11) |  |  |  |  |
|  | RA and FM | N= 25 | 42.6 (10.2) | 25 (100) | 9.5 (6.5) | DAS28 | 5.6 (1.1) | 12.3 (9.1) | 2.8 (3.2) | 64 (23.6) | 38.2 (16.8) |
|  |  |  |  |  |  | CDAI | 23.3 (12.1) |  |  |  |  |
| Vilaseca et al  Effect of the Coexistence of Fibromyalgia in the DAS28 Index in Women With Rheumatoid Arthritis (2008) (35) | RA only | N= 44 | 60.8 (15.1) | 44 (100) | 4.8 (4.5) | DAS28 | 3.39 (1.15) | 2.3 (5.1) | 0.8 (1.8) | 39.7 (26.6) | 28.1 (16.1) |
|  | RA and FM | N= 9 | 64.0 (14.4) | 9 (100) | 3.6 (3.5) | DAS28 | 5.55 (0.78) | 17.4 (9.5) | 1.1 (1.4) | 56.2 (21.0) | 30.8 (17.3) |
| Zammurrad et al  Disease Activity Score in Rheumatoid Arthritis with or without Secondary Fibromyalgia (2013) (37) | RA only | N=107 | 42.2 (12.3) | 97 (90.7) | 7.6 (6.7) | DAS28 | 3.9 (1.2) | 13.1 | 2.8 | 62.7 | 38.9 |
|  | RA and FM | N=31 | 45.4 (10.7) | 30 (96.8) | 5.3 (1.5) | DAS28 | 5.3 (1.5) | 1.7 | 4.1 | 38.0 | 30.7 |

**Supplementary Table S7: Comparison of DAS between patients with and without FM in the AS studies**

| Study | | Number of patients | Age, years, median (IQR)/mean (SD) | Females, n (%) | Disease Duration, years, median (IQR)/mean (SD) | Disease Activity Indices Used | DAS, Median (IQR)/ Mean (SD) |  |
| --- | --- | --- | --- | --- | --- | --- | --- | --- |
| **Ankylosing Spondylitis** | | | | | | | | |
| Almodóvar R et al.  Fibromyalgia in Patients with Ankylosing Spondylitis: Prevalence and Utility of the Measures of Activity, Function and Radiological Damage (2010) (1) | AS only | n=443 | 51 (13) | n= 107 (24) | 22 (13) | BASDAI | 3.7 (2.2) |  |
|  | AS & FM | n=19 | 48 (7) | n= 13 (68) | 21 (10) | BASDAI | 6.5 (2.2) |  |
| Aloush V et al.  Fibromyalgia in Women with Ankylosing Spondylitis (2007) (5) | AS only | n=27 | Not Stated | n=9 (33.3) | Not Stated | BASDAI | Values Not Stated but “the BASFI and BASDAI scores were significantly increased in patients who were diagnosed with FM” |  |
|  | AS & FM | n=9 | Not Stated | n=9 (100) | Not Stated | BASDAI |  |  |
| Azevedo VF et al.  Occurrence of Fibromyalgia in Patients with Ankylosing Spondylitis (2010) (7) | AS only | n=60 | Not Stated | n=11 (18.3) | Not Stated | BASDAI | 4.9 (2.38) |  |
|  | AS & FM | n=11 | Not Stated | n=6 (54.5) | Not Stated | BASDAI | 7.2 (1.61) |  |
| Demirdal et al  COEXISTING OF FIBROMYALGIA SYNDROME AND ANKYLOSING SPONDYLITIS (2013) (12) | AS only | N=64 | 38.12 (12.20) | N=8 (12.5) | 10.65 (9.09) | BASDAI | 3.35 (1.90) |  |
|  | AS & FM | N=13 | 37.15 (12.48) | 8:5 (38.5) | 9.46 (8.42) | BASDAI | 4.73 (2.13) |  |
| Haliloglu et al.  Fibromyalgia in Patients with Other Rheumatic Diseases: Prevalence and Relationship with Disease Activity (2014) (20) | AS only | n=104 | 40.13 (12.3) | n=44 (42.3) | Not Stated | BASDAI | 5.01 (1.4) |  |
|  | AS & FM | n=15 | 38.06 (9.07) | n=14 (93.3) | Not Stated | BASDAI | 7.26 (0.64) |  |
| **Other AxSpA** | | | | | | | | |
| Macfarlane et al  The co-occurrence and characteristics of patients with axial spondyloarthritis who meet criteria for fibromyalgia: results from a UK national register (BSRBR-AS) (27) | AxSpa alone | n= 1504 | Not stated | Not stated | Not stated | BASDAI | 3.6 95% CI 3.5–3.8 |  |
|  | AxSpa and FM | n= 311 | Not stated | Not stated | Not stated | BASDAI | 6.7 95% CI 6.5–6.9 |  |
| Salaffi F et al.  Fibromyalgia in Patients with Axial Spondylo-arthritis: Epidemiological Profile and Effect on Measures of Disease Activity (2014) (31) | AxSpA only | n=343 | 51.1 (12.2) | n=19 (17.1) | Not Stated | ASDAS-ESR | 3 (0.6) |  |
|  |  |  |  |  |  | ASDAS-CRP | 3.1 (0.7) |  |
|  |  |  |  |  |  | ASDAS (CRP+ESR+GH) | 3.3 (0.7) |  |
|  |  |  |  |  |  | ASDAS (CRP+ESR+fatigue) | 3.5 (0.7) |  |
|  |  |  |  |  |  | BASDAI: 4.8 (1.5) | 4.8 (1.5) |  |
|  |  |  |  |  |  | BASDAI 4 | 5.2 (1.4) |  |
|  |  |  |  |  |  | Mini BASDAI (w/o Q1, Q4) | 4.8 (1.7) |  |
|  |  |  |  |  |  | Mini BASDAI (w/o Q4) | 5.1 (1.5) |  |
|  |  |  |  |  |  | Mini BASDAI (w/o Q1) | 4.8 (1.5) |  |
|  |  |  |  |  |  | Mini BASDAI (w/o Q5, Q4) | 5 (1.5) |  |
|  |  |  |  |  |  | Mini BASDAI (w/o Q1, Q4, Q5) | 4.7 (1.7) |  |
|  | AxSpA & FM | n=59 | 52.9 (10.9) | n=34 (56.7) | Not Stated | ASDAS-ESR | 3.1 (0.7) |  |
|  |  |  |  |  |  | ASDAS-CRP | 3.2 (0.7) |  |
|  |  |  |  |  |  | ASDAS (CRP+ESR+GH) | 3.4 (0.7) |  |
|  |  |  |  |  |  | ASDAS (CRP+ESR+fatigue) | 3.5 (0.7) |  |
|  |  |  |  |  |  | BASDAI | 4.9 (1.7) |  |
|  |  |  |  |  |  | BASDAI 4 | 5.2 (1.9) |  |
|  |  |  |  |  |  | Mini BASDAI (w/o Q1, Q4) | 4.9 (1.8) |  |
|  |  |  |  |  |  | Mini BASDAI (w/o Q4) | 5 (1.9) |  |
|  |  |  |  |  |  | Mini BASDAI (w/o Q1) | 5 (1.8) |  |
|  |  |  |  |  |  | Mini BASDAI (w/o Q5, Q4) | 5 (1.9) |  |
|  |  |  |  |  |  | Mini BASDAI (w/o Q1, Q4, Q5) | 4.8 (1.9) |  |
| Watch et al  Fibromyalgia in Spondyloarthritis: Effect on Disease  Activity Assessment in Clinical Practice (36) | axSpA alone | n=69 | 40.9 (17.7) IQR | 18 (26.1) IQR | 11 (13) IQR | BASDAI  ASDAS-CRP | BASDAI 2.2 (3.2) IQR  ASDAS-CRP 2 (1.3) IQR |  |
|  | axSpA and FM | n=12 | 44.6 (23.0) IQR | 6 (50.0) IQR | 6.5 (9) IQR | BASDAI  ASDAS-CRP | BASDAI 3.2 (5.4) IQR  ASDAS-CRP 2.5 (2.3) IQR |  |

Axial-SpA includes patients with either AS or psoriatic arthritis characterised by axial invovlement; AxSpa: Axial Spondyloarthritis; ASDAS: Ankylosing Spondylitis Disease Activity Index; GH: Global Health

Supplementary Table S8: Comparison of Disease Activity Scores between Patients With and Without FM in PsA studies

|  | | | | | | |
| --- | --- | --- | --- | --- | --- | --- |
| Study | Number of Patients | Age in years; Median (IQR)/ Mean (SD)/ Mean | Females, n (%) | Disease Duration in years (unless stated); Median (IQR)/ Mean (SD) | Disease Activity Indices Used | Disease Activity Score |
|  |  |  |  |  |  | DAS, median (IQR)/ mean (SD)/ mean |
| **Psoriatic arthritis** | | | | | | |
| Brikman et al  The Effect of the Presence of Fibromyalgia on Common Clinical Disease Activity Indices in Patients with Psoriatic Arthritis: A Cross-sectional Study (2016) (6) | PsA alone = 60 | 50.8 (14.3) | 30 (50) | 10 (7.3) | CPDAI | 4.25 (3.14) |
|  |  |  |  |  | MDA | 26 (43.3%) |
|  |  |  |  |  | DAPSA | 12.82 (12.7) |
|  |  |  |  |  | DAS28 | 2.49 (1.18) |
|  |  |  |  |  | PASI | 2.1 (0.2–6.3) |
|  |  |  |  |  | BASDAI | 2.87 (2.35) |
|  |  |  |  |  | LEI | 0 (0–1) |
|  | PsA and FM=13 | 55.8 (7.2) | 12 (92.3) | 11.3 (8.8) | CPDAI | 9.23 (1.92) |
|  |  |  |  |  | MDA | 0 |
|  |  |  |  |  | DAPSA | 27.53 (19.23) |
|  |  |  |  |  | DAS28 | 3.39 (0.93) |
|  |  |  |  |  | PASI | 3 (0.8–8.6) |
|  |  |  |  |  | BASDAI | 7.18 (1.73) |
|  |  |  |  |  | LEI | 3 (2–4) |
| Graceffa et al  Clinical remission of psoriatic arthritis in patients receiving continuous biological therapies for 1 year: the experience of an outpatient dermatological clinic for psoriasis (2015) (19) | PsA alone= 62 | Not stated | Not stated | Not stated | Clinical remission | Association with remission:  HR = 11.71, 95% CI 1.61–85.22 |
|  | PsA and FM= 12 | Not stated | Not stated | Not stated | Clinical remission | See above |

^a^CPDAI = Composite Psoriatic Disease Activity Index, ^b^MDA= Minimal Disease Activity, ^c^DAPSA= Disease Activity Index for Psoriatic Arthritis, ^d^DAS28= Disease Activity Score of 28 joints, ^e^PASI: Psoriasis Activity and Severity Index. ^f^BASDAI = Bath Ankylosing Spondylitis Disease Activity Index ^g^LEI= Leeds Enthesitis Index.

**Supplementary Figure S1. Random-effects meta-analysis of prevalence figures for FM in RA**

**Supplementary Figure S2. Random-effects meta-analysis of prevalence figures for FM in AS**

**Supplementary Figure S3. Random-effects meta-analysis of prevalence figures for FM in PsA**

**References**

1. Almodovar R, Carmona L, Zarco P, Collantes E, Gonzalez C, Mulero J, et al. Fibromyalgia in patients with ankylosing spondylitis: prevalence and utility of the measures of activity, function and radiological damage. Clin Exp Rheumatol. 2011/02/18. 2010;28(6 Suppl 63):S33-9.

2. Amity CL, Schlenk EA, Gold KN, Eckels MM, Mohan N, Balasubramani GK, et al. Agreement of Physicians and Nurses Performing Tender and Swollen Joint Counts in Rheumatoid Arthritis. Jcr-Journal Clin Rheumatol. 2016;22(1):30–4.

3. Abbasi L, Haidri FR. Fibromyalgia complicating disease management in rheumatoid arthritis. J Coll Physicians Surg Pak. 2014/06/24. 2014;24(6):424–7.

4. Ahmad J, Blumen H, Tagoe CE. Association of antithyroid peroxidase antibody with fibromyalgia in rheumatoid arthritis. Rheumatol Int. 2015/05/16. 2015;35(8):1415–21.

5. Aloush V, Ablin JN, Reitblat T, Caspi D, Elkayam O. Fibromyalgia in women with ankylosing spondylitis. Rheumatol Int. 2007/05/04. 2007;27(9):865–8.

6. Brikman S, Furer V, Wollman J, Borok S, Matz H, Polachek A, et al. The Effect of the Presence of Fibromyalgia on Common Clinical Disease Activity Indices in Patients with Psoriatic Arthritis: A Cross-sectional Study. J Rheumatol [Internet]. 2016; Available from: http://www.jrheum.org/cgi/doi/10.3899/jrheum.151491%5Cnhttp://www.ncbi.nlm.nih.gov/pubmed/27252430

7. Azevedo VF, Paiva Edos S, Felippe LR, Moreira RA. Occurrence of fibromyalgia in patients with ankylosing spondylitis. Rev Bras Reum. 2011/01/19. 2010;50(6):646–50.

8. Buyukbese M, Pamuk O, Yurekli O, Yesil N. Effect of fibromyalgia on bone mineral density in patients with fibromylagia and rheumatoid arthritis. J Postgrad Med. 2013;59(2):106.

9. Chakr RM da S, Brenol C, Ranzolin A, Bernardes A, Dalosto AP, Ferrari G, et al. Rheumatoid arthritis seems to have DMARD treatment decision influenced by fibromyalgia. Rev Bras Reumatol. 2016;(x x):1–9.

10. Christensen AW, Rifbjerg-Madsen S, Christensen R, Dreyer L, Tillingsøe H, Seven S, et al. Non-nociceptive pain in rheumatoid arthritis is frequent and affects disease activity estimation: cross-sectional data from the FRAME study. Scand J Rheumatol. 2016;9742(April):1–9.

11. Coury F, Rossat A, Tebib A, Letroublon MC, Gagnard A, Fantino B, et al. Rheumatoid arthritis and fibromyalgia: a frequent unrelated association complicating disease management. J Rheumatol. 2009/01/10. 2009;36(1):58–62.

12. Demirdal S, Çakir T, Tugrul T, Subaşi V. COEXISTING OF FIBROMYALGIA SYNDROME AND ANKYLOSING SPONDYLITIS.

13. Di Carlo M, Becciolini A, Lato V, Crotti C, Favalli EG, Salaffi F. The 12-item Psoriatic Arthritis Impact of Disease Questionnaire: Construct Validity, Reliability, and Interpretability in a Clinical Setting. J Rheumatol. 2017;44(3):279–85.

14. Dhir V, Lawrence A, Aggarwal A, Misra R. Fibromyalgia is common and adversely affects pain and fatigue perception in North Indian patients with rheumatoid arthritis. J Rheumatol. 2009;36(11):2443–9.

15. El-Rabbat M S, Mahmoud NK, Gheita TA. Clinical significance of fibromyalgia syndrome in different rheumatic diseases: Relation to disease activity and quality of life. Reumatol Clin. 2017;in press.

16. Fan A, Pereira B, Tournadre A, Tatar Z, Malochet-Guinamand S, Mathieu S, et al. Frequency of concomitant fibromyalgia in rheumatic diseases: Monocentric study of 691 patients. Semin Arthritis Rheum. 2017;47(1):129–32.

17. Gheita TA, Sayed S, Gheita HA, Kenawy SA. Vitamin D status in rheumatoid arthritis patients: relation to clinical manifestations, disease activity, quality of life and fibromyalgia syndrome. Int J Rheum Dis. 2014;294–9.

18. Gist AC, Guymer EK, Eades LE, Leech M, Littlejohn GO. Fibromyalgia remains a significant burden in rheumatoid arthritis patients in Australia. International Journal of Rheumatic Diseases. 2017;

19. Graceffa D, Maiani E, Sperduti I, Ceralli F, Bonifati C. Clinical remission of psoriatic arthritis in patients receiving continuous biological therapies for 1 year: The experience of an outpatient dermatological clinic for psoriasis. Clin Exp Dermatol. 2015;40(2):136–41.

20. Haliloglu S, Carlioglu A, Akdeniz D, Karaaslan Y, Kosar A. Fibromyalgia in patients with other rheumatic diseases: prevalence and relationship with disease activity. Rheumatol Int. 2014/03/05. 2014;34(9):1275–80.

21. Husted JA, Tom BDM, Farewell VT, Gladman DD. Longitudinal analysis of fatigue in psoriatic arthritis. J Rheumatol. 2010;37(9):1878–84.

22. Joharatnam N, McWilliams DF, Wilson D, Wheeler M, Pande I, Walsh DA. A cross-sectional study of pain sensitivity, disease-activity assessment, mental health, and fibromyalgia status in rheumatoid arthritis. Arthritis Res Ther. 2015/01/21. 2015;17:11.

23. Kapoor SR, Hider SL, Brownfield A, Mattey DL, Packham JC. Fibromyalgia in patients with rheumatoid arthritis: driven by depression or joint damage? Clin Exp Rheumatol. 2012/03/14. 2011;29(6 Suppl 69):S88-91.

24. Karpouzas GA, Dolatabadi S, Moran R, Li N, Nicassio PM, Weisman MH. Correlates and predictors of disability in vulnerable US Hispanics with rheumatoid arthritis. Arthritis Care Res (Hoboken). 2012/04/11. 2012;64(9):1274–81.

25. Lage-Hansen PR, Chrysidis S, Lage-Hansen M, Hougaard A, Ejstrup L, Amris K. Concomitant fibromyalgia in rheumatoid arthritis is associated with the more frequent use of biological therapy: a cross-sectional study. Scand J Rheumatol. 2015;9742(September):1–4.

26. Lee YC, Bingham CO, Edwards RR, Marder W, Kristine P, Bolster MB, et al. Pain Sensitization is Associated with Disease Activity in Rheumatoid Arthritis Patients: A Cross-Sectional Study. Arthritis Care Res (Hoboken). 2017;

27. Macfarlane GJ, Barnish MS, Pathan E, Martin KR, Haywood KL, Siebert S, et al. The co-occurrence and characteristics of patients with axial spondyloarthritis who meet criteria for fibromyalgia: Results from a UK national register (BSRBR-AS). Arthritis Rheumatol (Hoboken, NJ). 2017;

28. Mian AN, Chaabo K, Wajed J, Subesinghe S, Gullick NJ, Kirkham B, et al. Rheumatoid arthritis patients with fibromyalgic clinical features have significantly less synovitis as defined by power Doppler ultrasound. BMC Musculoskelet Disord. 2016;17:404.

29. Pollard LC, Ibrahim F, Choy EH, Scott DL. Pain thresholds in rheumatoid arthritis: The effect of tender point counts and disease duration. J Rheumatol. 2012;39(1):28–31.

30. Ranzolin A, Brenol JC, Bredemeier M, Guarienti J, Rizzatti M, Feldman D, et al. Association of concomitant fibromyalgia with worse disease activity score in 28 joints, health assessment questionnaire, and short form 36 scores in patients with rheumatoid arthritis. Arthritis Rheum Arthritis Care Res. 2009;61(6):794–801.

31. Salaffi F, De Angelis R, Carotti M, Gutierrez M, Sarzi-Puttini P, Atzeni F. Fibromyalgia in patients with axial spondyloarthritis: epidemiological profile and effect on measures of disease activity. Rheumatol Int. 2014/02/11. 2014;34(8):1103–10.

32. Salaffi F, Gerardi MC, Atzeni F, Batticciotto A, Talotta R, Draghessi A, et al. The influence of fibromyalgia on achieving remission in patients with long-standing rheumatoid arthritis. Rheumatol Int. 2017;37(12):2035–42.

33. Ton E, Bakker MF, Verstappen SMM, Ter Borg EJ, Van Albada-Kuipers IA, Schenk Y, et al. Look beyond the disease activity score of 28 joints (DAS28): Tender points influence the DAS28 in patients with rheumatoid arthritis. J Rheumatol. 2012;39(1):22–7.

34. Urrows S, Affleck G, Tennen H, Higgins P. Unique clinical and psychological correlates of fibromyalgia tender points and joint tenderness in rheumatoid arthritis. Arthritis Rheum. 1994;37(10):1513–20.

35. Roig Vilaseca D, Hoces Otero C. Effect of the Coexistence of Fibromyalgia in the DAS28 Index in Women With Rheumatoid Arthritis. Reumatol Clínica (English Ed. 2008;4(3):96–9.

36. Wach J, Letroublon M-C, Coury F, Tebib JG. Fibromyalgia in Spondyloarthritis: Effect on Disease Activity Assessment in Clinical Practice. J Rheumatol. 2016;43(11):2056–63.

37. Zammurrad S, Aziz W, Farooqi A. Disease activity score in rheumatoid arthritis with or without secondary fibromyalgia. J Coll Physicians Surg Pakistan. 2013;23(6):413–7.

38. Da Silva Chakr RM, Brenol JCT, Behar M, Mendonça JA, Kohem CL, Monticielo OA, et al. Is ultrasound a better target than clinical disease activity scores in Rheumatoid Arthritis with fibromyalgia? A case-control study. PLoS One. 2015;10(3).

39. Nawito Z, Rady HM, Maged LA. The impact of fibromyalgia on disease assessment in rheumatoid arthritis patients. Egypt Rheumatol. 2013;35(3):115–9.

40. Ghib LJ, Tamas M-M, Damian LO, Felea I, Muntean LM, Rednic N, et al. The role of ultrasonography in assessing disease activity in patients with rheumatoid arthritis and associated fibromyalgia. Med Ultrason. 2015;17(3):339–44.

41. Pollard LC, Kingsley GH, Choy EH, Scott DL. Fibromyalgic rheumatoid arthritis and disease assessment. Rheumatology (Oxford). 2010;49(5):924–8.
